# Bibliometric analysis of highly cited articles on ecosystem services

**DOI:** 10.1371/journal.pone.0210707

**Published:** 2019-02-11

**Authors:** Xinmin Zhang, Ronald C. Estoque, Hualin Xie, Yuji Murayama, Manjula Ranagalage

**Affiliations:** 1 Graduate School of Life and Environmental Sciences, University of Tsukuba, Tsukuba, Japan; 2 Center for Social and Environmental Systems Research, National Institute for Environmental Studies, Tsukuba, Japan; 3 Institute of Ecological Civilization, Jiangxi University of Finance and Economics, Nanchang, China; 4 Faculty of Life and Environmental Sciences, University of Tsukuba, Tsukuba, Japan; 5 Faculty of Social Sciences and Humanities, Rajarata University of Sri Lanka, Mihintale, Sri Lanka; Universita degli Studi di Genova, ITALY

## Abstract

This paper presents global research trends involving highly cited articles on ecosystem services from 1981 to 2017 based on a bibliometric analysis of such articles from the SCI-E and SSCI databases of the Web of Science. The analysis revealed that there were 132 highly cited articles, most of which were published between 2005 and 2014. Based on author keywords, the term ecosystem services was strongly linked to biodiversity. The top three journals in terms of total number of highly cited articles published were *Ecological Economics*, *PNAS*, and *Ecological Indicators*. Despite ranking sixth overall, *Science* ranked first in both impact factor and total citations per article. The US, UK, Netherlands, Spain, and Sweden were the top five most productive and cooperative countries in the world based on total number of highly cited articles and co-authorship network, respectively. The US was highly connected to Canada, the Netherlands, China and the UK. Stockholm University and Stanford University were the most productive institutions in Europe and North America, respectively. Stanford University is associated with many scholars in the field of ecosystem services research because of the InVEST model. Robert Costanza was the most prolific and highly cited author, the latter being largely due to the first valuation of the world’s ecosystem services and natural capital, he and his co-authors published in 1997 in *Nature*. Terrestrial, urban, and forest ecosystems were the top types of ecosystems assessed. Regulating and provisioning services were the major ecosystem services studied. Quantitative and qualitative assessments were the main research focus. Most of these highly cited studies on ecosystem services are done on areas geographically located in North America and Europe.

## Introduction

Intensified and continuously expanding human activities are affecting the Earth’s natural ecosystems, known as the human life support systems [1], including the ecosystem services they provide to the people and society [2]. Ecosystem services are classified into provisioning, regulating, supporting, and cultural services [3]. These services generally play important roles in maintaining human well-being, both directly and indirectly. However, these services have been affected by various anthropogenic factors. For instance, based on earlier studies [4–6], Costanza et al. [7] have indicated that due to land use change between 1997 and 2011, the value of global ecosystem services had declined by $4.3–20.2 trillion per year. Reports have also indicated that approximately 60% of all ecosystem services have been degraded or used unsustainably [3]. This is alarming because the loss and degradation of these services can significantly impact human well-being and pose risks to regional and global eco-security [3,8]. Various case studies at the regional and local levels have also illustrated and confirmed that rapid and unplanned urbanization can affect the provisions of ecosystem services [9–12].

Over the past decade or so, the ecosystem services concept has gained attention from various global initiatives, including the Millennium Ecosystem Assessment (MEA) (www.millenniumassessment.org), The Economics of Ecosystems and Biodiversity (TEEB) (www.teebweb.org), Intergovernmental Platform on Biodiversity and Ecosystem Services (IPBES) (www.ipbes.net), and the Ecosystem Services Partnership (ESP) (www.es-partnership.org). It has also been highlighted in the most recent EU Biodiversity Strategy to 2020 (ec.europa.eu/environment/nature/biodiversity/strategy/index_en.htm) and the United Nations Sustainable Development Goals (UN SDGs) (e.g., SDG 15) (sustainabledevelopment.un.org). All these initiatives help promote and advance ecosystem services research towards sustainable development.

The field of ecosystem services research is relatively new. The term “ecosystem services” was first introduced in 1981 [13], and was later popularized as a concept through efforts such as the MEA [14–15]. And despite being relatively new, there is a rich and rapidly growing body of literature concerning ecosystem services, with several qualitative reviews having been performed, including those related to the impacts of climate change on ecosystem services and biodiversity [16–18]. Quantitative literature reviews on ecosystem services are also available at the country level (e.g., China, New Zealand, and Argentina) [19–21]. Bibliometric reviews regarding ecosystem services research and assessments have also been performed at both the regional (e.g., Africa, Asia, and Latin America) [22–24] and global [25–27] levels.

Generally, a bibliometric analysis is performed to evaluate both research trends and scholarly networks in different research disciplines [28–32]. This type of analysis can also provide guidance to young and budding researchers [31]. It can also encourage and challenge researchers to conduct further studies [32–34]. Other reviews have combined a bibliometric analysis with a systematic and/or conceptual-theoretical review (e.g., [18,32]). However, in this study we focus only on the bibliometric analysis of ecosystem services based on highly cited articles. In most cases, a bibliometric analysis summarizes research trends and scholarly networks based on publication outputs, subject categories, major journals, active authors, productive countries, research institutions, and keyword frequencies [30–32, 35].

Previous bibliometric studies were conducted to reveal the characteristics of highly cited articles on specific topics, such as species distribution predictive models [36], the Antarctic [37], environmental science [38], and the subject category of horticulture [39]. Highly cited articles are typically authored by a large number of researchers and often involve international collaboration [40]. They are considered to be classic works that have significant impacts on their respective subjects in a worldwide context [39]. It is therefore necessary to explore highly cited articles as opposed to those that are less frequently cited. However, there is still no standard way to identify highly cited articles. One way is based on citations rates or thresholds, and another way is by choosing a specific number of articles in the top of the list of highly cited works (i.e., to set a concrete number of articles or to set a percentage of articles (e.g., top 1%)) [41]. In other works, a citation threshold of > 100 times has been used and referred to as highly cited articles [36–39]. In this study, we also used this citation threshold.

There is still a lack of comprehensive bibliometric analyses focusing on ecosystem services research at the global level that also consider scholarly networks. Hence, this study aimed at filling this gap by examining the global trends and scholarly networks involving ecosystem services research based on a bibliometric analysis of highly cited articles from 1981 to 2017 found in the SCI-E and SSCI databases of the Web of Science. The period of analysis was decided to capture the development and progress in ecosystem services research since its introduction in 1981 [13]. The analysis was performed based on publication outputs, author keywords, journals, institutions, authors, and countries. Ecosystem types and services, research focuses, and case study sites were also considered and reviewed.

## Materials and methods

Most bibliometric studies have been conducted based on the Web of Science [31–32, 42–43]. According to the 2018 Journal Citation Reports (JCR), the ISI Web of Science indexes 11,655 major journals with citation references across 234 scientific disciplines [44]. On March 10, 2018, we used the ISI Web of Science database to gather academic publications on ecosystem services from 1981 to 2017. We searched all publications related to ecosystem services research from the ISI Web of Science Core Collection database (SCI-E and SSCI) using the search terms “ecosystem service” OR “ecosystem services” in the “Title” field. In the “document type” field, our search was filtered for articles only (other document types such as reviews and meeting abstracts were excluded), written and published in English up until the end of 2017. This filtering resulted to 3,057 articles. Based on previous research, articles that were cited more than 100 times were generally referred to as highly cited [36–38]. By employing the same threshold, we retrieved 132 highly cited research articles. This accounts for 4.32% of the total articles. Important bibliometric information were subsequently retrieved, including author name(s), author affiliation(s), subject category(ies), journal name(s), publication title(s), and publication year(s). For this study’s purpose, articles originating from England, Scotland, Wales, and Northern Ireland were classified as articles from the United Kingdom (UK) [42]. We collected the impact factor (IF) of each journal from the 2018 JCR.

We performed our bibliometric analysis using a quantitative analysis approach and knowledge mapping technique. The quantitative analysis was performed based on the information provided by Web of Science. Knowledge mapping (i.e., network analysis) was performed using VOSviewer (www.vosviewer.com), in which we focused on the network and “link strength” between author keywords, countries, institutions, and authors. A network analysis is usually performed to map the scope and structure of the discipline while discovering key research clusters [45]. A recent comparative study indicated that fractional counting is preferable to full counting [46]. The fractional counting approach, which assigns co-authored publications fractionally to each author, does provide proper field-normalized results [47]. On this basis, we used fractional counting in our analysis. This process produced the following results: (i) the co-occurrence network of the most frequently used author keywords, (ii) co-authorship network of the top countries, (iii) co-authorship network of the top institutions, and (iv) co-authorship network of the most productive authors.

In the analysis of the characteristic of highly cited articles on ecosystem services, we only considered articles that clearly indicate the study area (i.e., local, regional, national, and continental scale), and there were 71 articles remained out of the 132 highly cited articles for the rest of the analysis. In addition, the type of ecosystem, ecosystem service being assessed, the focus of the research, and those that clearly mentioned their case study site among the 71 articles were summarized and reviewed.

## Results and discussion

### Highly cited articles, most active journals, and author keywords

**Fig 1:**
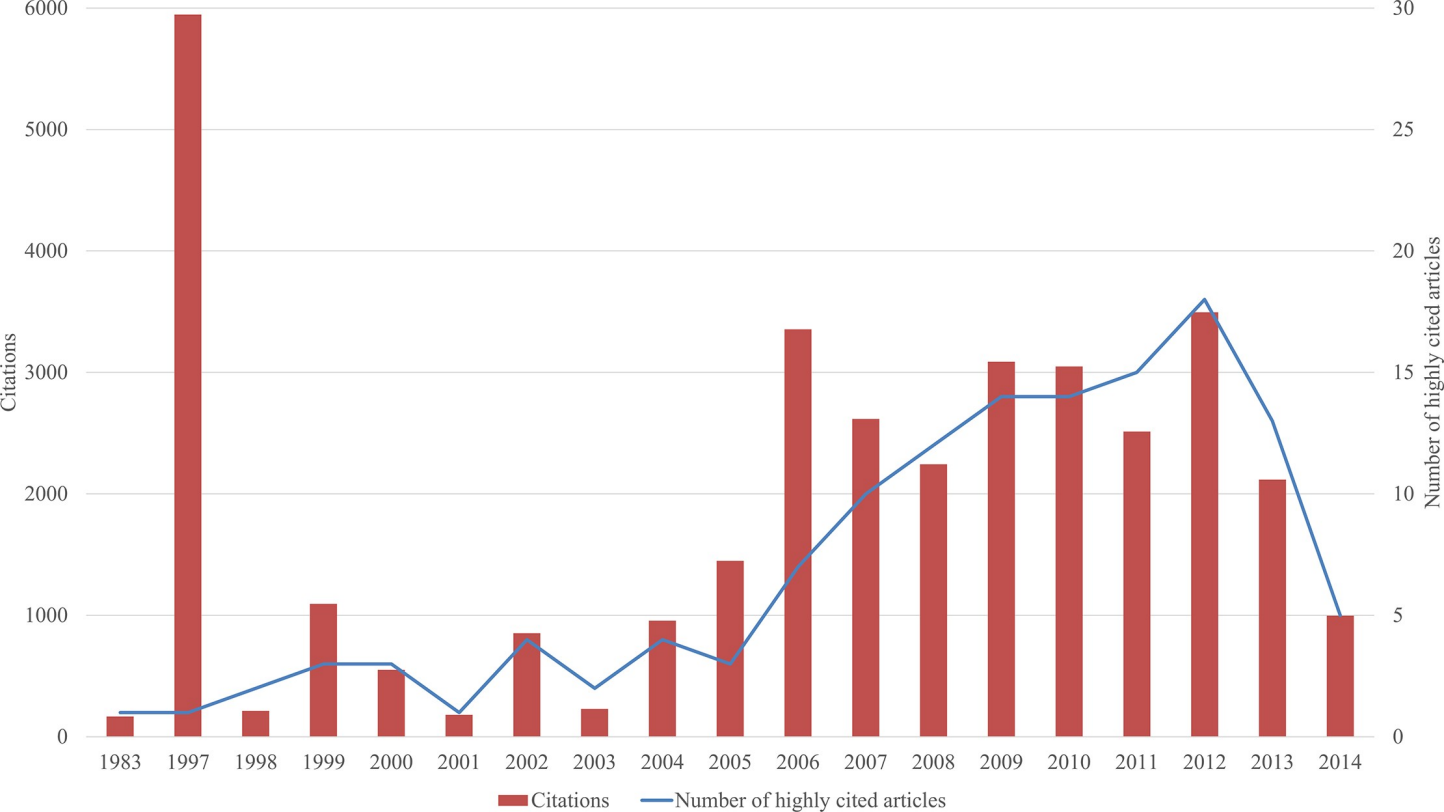
Highly cited articles involving ecosystem services and the number of times they were cited across years.

Fig 1 shows the temporal distribution of the 132 highly cited articles. Results revealed two quite different growth periods of highly cited articles over the past 30+ years. There were only 24 highly cited articles related to ecosystem services prior to 2005 (including 2005). However, after 2005, the cumulative number of highly cited articles increased dramatically to 108, tallying an average increase of 12 articles per year. Notably, the final MEA report was published in 2005 [3], and the most recent TEEB report was published in 2010 [48]. We consider these reports to have been instrumental in establishing the ecosystem services concept at the forefront of human-environmental studies. This resulted in the development of global research interest and increased popularity among researchers and scientists. Our results are consistent with other previous observations that the ecosystem services concept has increasingly gained attention at the global level while becoming a top research area in environmental fields [8, 25, 49].

There were more than 1,400 citations for each year of the highly cited articles from 2005 to 2013. This is due to the combined effect of the increasing number of highly cited articles and the accumulation period. On the other hand, there was a decreasing trend involving total number of citations because most newly published articles (e.g., 2012–2014) had not been cited much at the time of our bibliometric research. Moreover, none was published after 2014 (2015–2017). Up until the time of our bibliometric search, the articles published during the period of 2015–2017 still did not have enough citations (> 100 times) to be called highly cited articles. Notably, only one highly cited article was published in 1997. This article examined the value of the world’s ecosystem services and natural capital [4] and has been cited approximately 6,000 times as of the end of 2017, according to Web of Science. The number of ecosystem services-related articles has been increasing exponentially, a trend which will likely continue [25, 49]. It is therefore probable that the number of highly cited articles and their citations will also increase at a similar rate.

Table 1 presents some bibliometric details of the top six journals with highly cited articles (i.e., those containing four or more articles) related to ecosystem services research. These are *Ecological Economics* (27), *PNAS* (15), *Ecological Indicators* (6), *BioScience* (5), *Ecology and Society* (4), and *Science* (4). These journals account for 46.21% of all highly cited articles used in this study.

**Table 1:** Top journals with the highly cited articles (more than 4 articles) in ecosystem services research (1981–2017).

Journal	NA	NC	NC/NA	IF (2017)	Web of Science category	Position
Ecological Economics	27	7101	263	3.895	Ecology	31 of 160, Q1
					Economics	21 of 353, Q1
					Environmental Sciences	52 of 242, Q1
					Environmental Studies	14 of 109, Q1
PNAS	15	4166	278	9.504	Multidisciplinary Sciences	5 of 64, Q1
Ecological Indicators	6	992	165	3.983	Environmental Sciences	49 of 242, Q1
BioScience	5	868	174	5.876	Biology	5 of 85, Q1
Ecology and Society	4	693	173	3.256	Ecology	45 of 160, Q2
					Environmental Studies	23 of 109, Q1
Science	4	3208	802	41.058	Multidisciplinary Sciences	2 of 64, Q1

NA: number of highly cited articles; NC: number of citations; IF: impact factor (JCR 2018). Q1 means the journal ranking top 25%, Q2 means the journal ranking 25%-50%.

*Ecological Economics* accounts for 27 highly cited articles (i.e., 20.45% of the total), and has an impact factor of 2.965 in 2017. It is not only the most productive journal, but also contains the highest number of total citations (Table 1). *Ecological Economics* was established in 1989 and focuses on publishing transdisciplinary research connecting ecology and economics [25]. As Costanza et al. [50] found, from the period of 2004–2014, approximately half of the most highly cited articles discuss ecological economics, which indicates the growing importance of ecosystem services in this journal. *Science* ranked sixth based on the total number of highly cited articles published. However, it ranked first in terms of total number of citations per article. Overall, results indicated that highly cited articles related to ecosystem services in *Science*, *PNAS*, and *Ecological Economics* significantly contributed to their respective impact factors (Table 1). Not surprisingly, the *Ecosystem Services* journal is not listed in Table 1. Established in 2012, *Ecosystem Services* is a relatively new journal. Despite this, there is a strong indication of its growing leadership role on ecosystem services research [25]. More highly cited articles will most likely come from this journal in the future because it is dedicated to promote the publications of ecosystem services research.

The major subject categories among the top six journals are related to environmental science and ecology (Table 1). A bibliometric analysis based on ecosystem services articles in the Scopus database also revealed that environmental science was at the top category [25]. The quartile ranking of these top journals is mainly referred to as Q1, which means the journal rank top 25% of Web of Science. These top journals support and cultivate research articles related to environmental science and ecology. The journals identified as most active were highlighted in a separate review [25].

Transdisciplinary journals (e.g., *Ecological Economics*, *PNAS*, and *Science*) have published a variety of research from various disciplines, and this arguably is responsible for their relatively high number of citations per article (Table 1). These journals have a high potential to absorb multidisciplinary research, including ecosystem services and its conceptual advancement. Needless to say, ecosystem services is, by nature, a multidisciplinary concept [25], and a rapidly emerging field of transdisciplinary scholarship [26].

**Fig 2:**
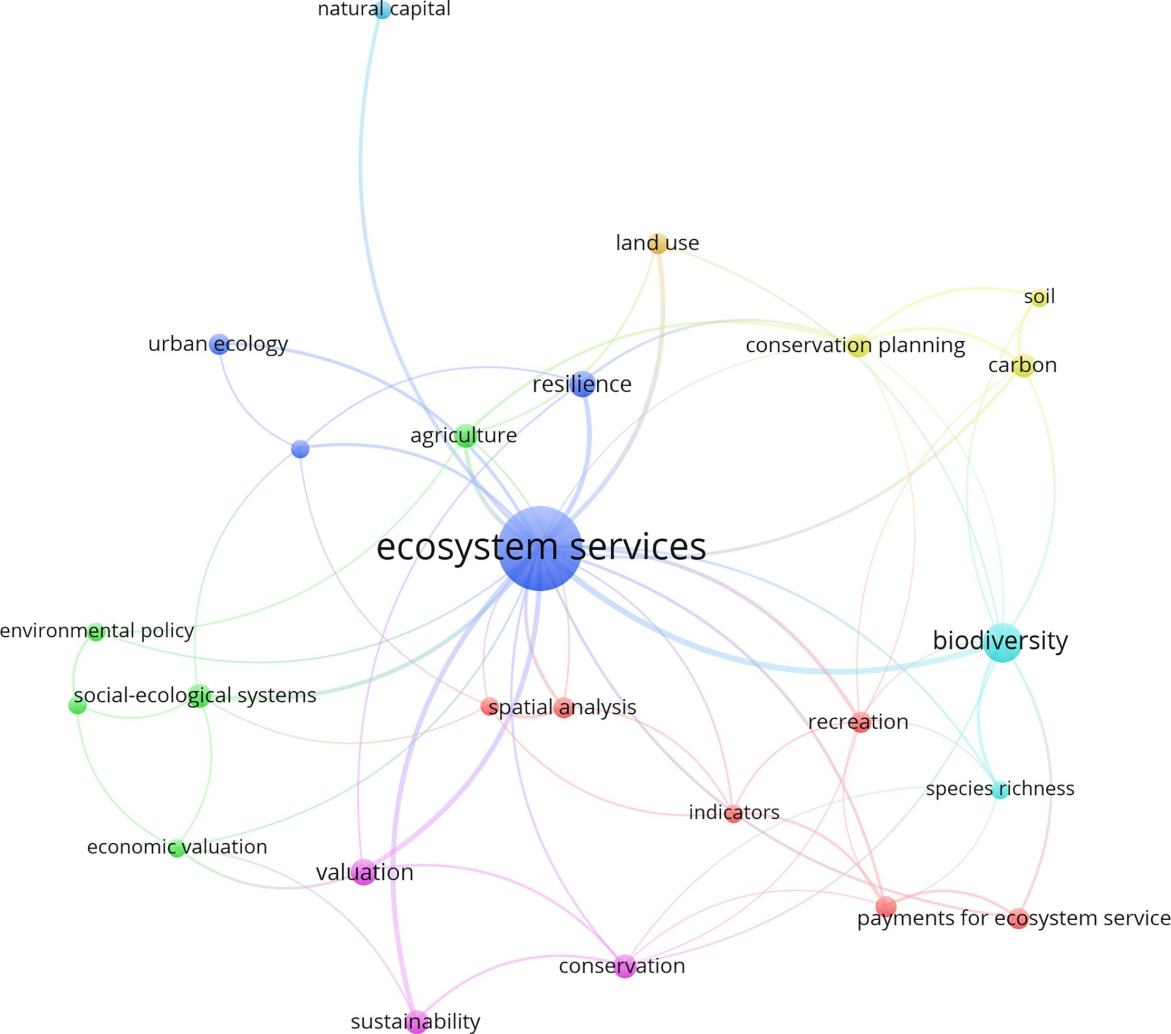
Co-occurrence network of the most frequently used author keywords. Note: A threshold of 3 was applied for these 132 highly cited articles, which resulted in a total of 25 keywords. The bubble size refers to the total number of highly cited articles, while line thickness and color refer to link strength and clustering, respectively.

**Fig 3:**
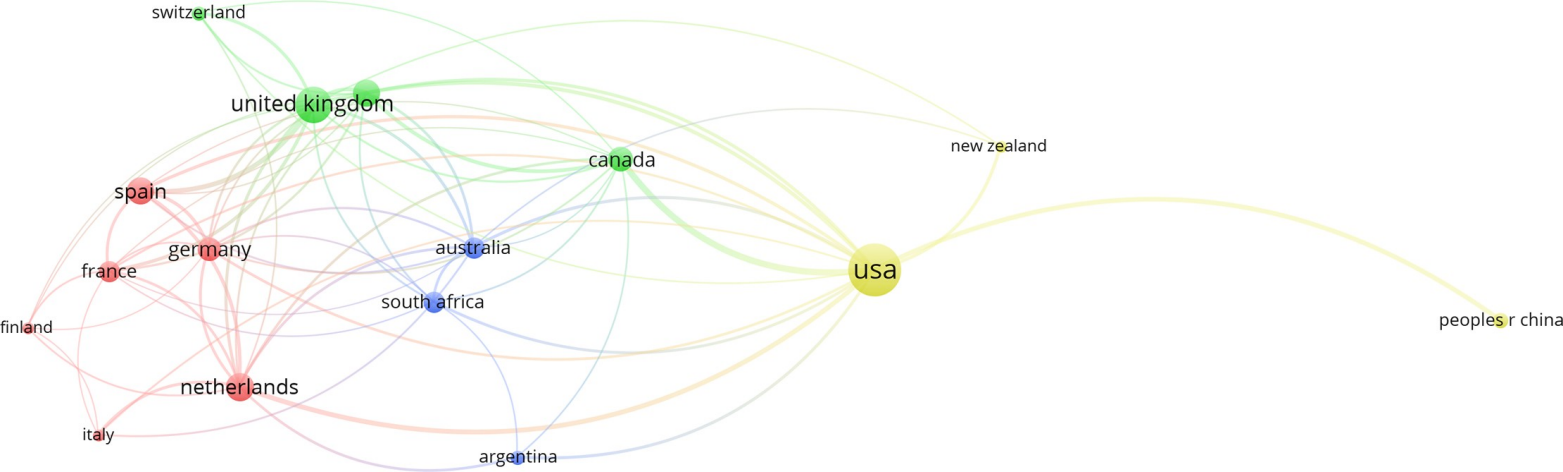
Co-authorship network of the top countries based on the total number of highly cited articles. Note: A threshold of 4 was applied for these 132 highly cited articles, which resulted in a total of 16 countries. The bubble size refers to the total number of highly cited articles, while line thickness and color refer to link strength and clustering, respectively.

The top author keywords can give indications of the research priorities and interests of scientists and researchers in the field of ecosystem services research. According to these keywords, the top five in terms of total occurrences were ecosystem services (57), biodiversity (13), valuation (6), resilience (6), and conservation planning (5). Ecosystem services had the highest link strength among all author keywords and was highly connected to biodiversity (Fig 2). The strength of the network between ecosystem services and biodiversity indicates a close relationship between these two concepts. In fact, there is still high interest among many researchers regarding this link [1, 51–54]. Researchers are also concerned with the way ecosystem services can be effectively managed and conserved, as indicated by the strong network between ecosystem services, valuation, and sustainability (Fig 3).

In this study, the keyword “landscape” did not appear in author keywords network (Fig 3). In ecosystem services research, a landscape can be referred to as either natural or cultural. There is still a need to expand the scope of ecosystem services research involving “landscape” to include both types. The keywords “land use”, “agriculture”, and “urban ecology” are related to natural landscapes. However, there are very few keywords presented and associated with cultural landscapes in the network. It is also worth noting that some more complex and emerging concepts (e.g., “trade-off”, and “synergy”) are still less popular among ecosystem services scholars. However, since these concepts are important to the study of ecosystem services [55–57], it is expected that they will gain more attention in future studies. Unsurprisingly, the keyword “sustainability” was also frequently used. Although it may have only been part of the main subject, this keyword has been explicitly used in many articles.

### The most active countries, institutions, and authors

The US, UK, Netherlands, Spain, and Sweden were among the top five countries in the world in terms of total number of highly cited articles published from 1981–2017 (Fig 3). Only one Asian country (i.e., China), one African country (i.e., South Africa), and one Latin American country (i.e., Argentina) were among the most productive countries worldwide. The US had the highest number of highly cited articles (70) within the study period, accounting for 23,477 of total citations. This indicates that the US is leading in ecosystem services research.

The co-authorship network presented in Fig 3 reflects the state of collaboration between the most productive countries. The US was highly connected to Canada, the Netherlands, China, and the UK. The total link strength between these four countries and the US accounted for 55.97% of the US’ total link strength. This shows that a substantial proportion of US’ network involves these four countries. The results enabled the identification of four clusters, as follows: countries surrounding the US (the yellow cluster), countries surrounding the UK (the green cluster), countries surrounding the Netherlands (the red cluster), and countries surrounding South Africa/Australia (the blue cluster) (Fig 3). Notably, the countries surrounding Netherlands mostly come from Western Europe.

**Fig 4:**
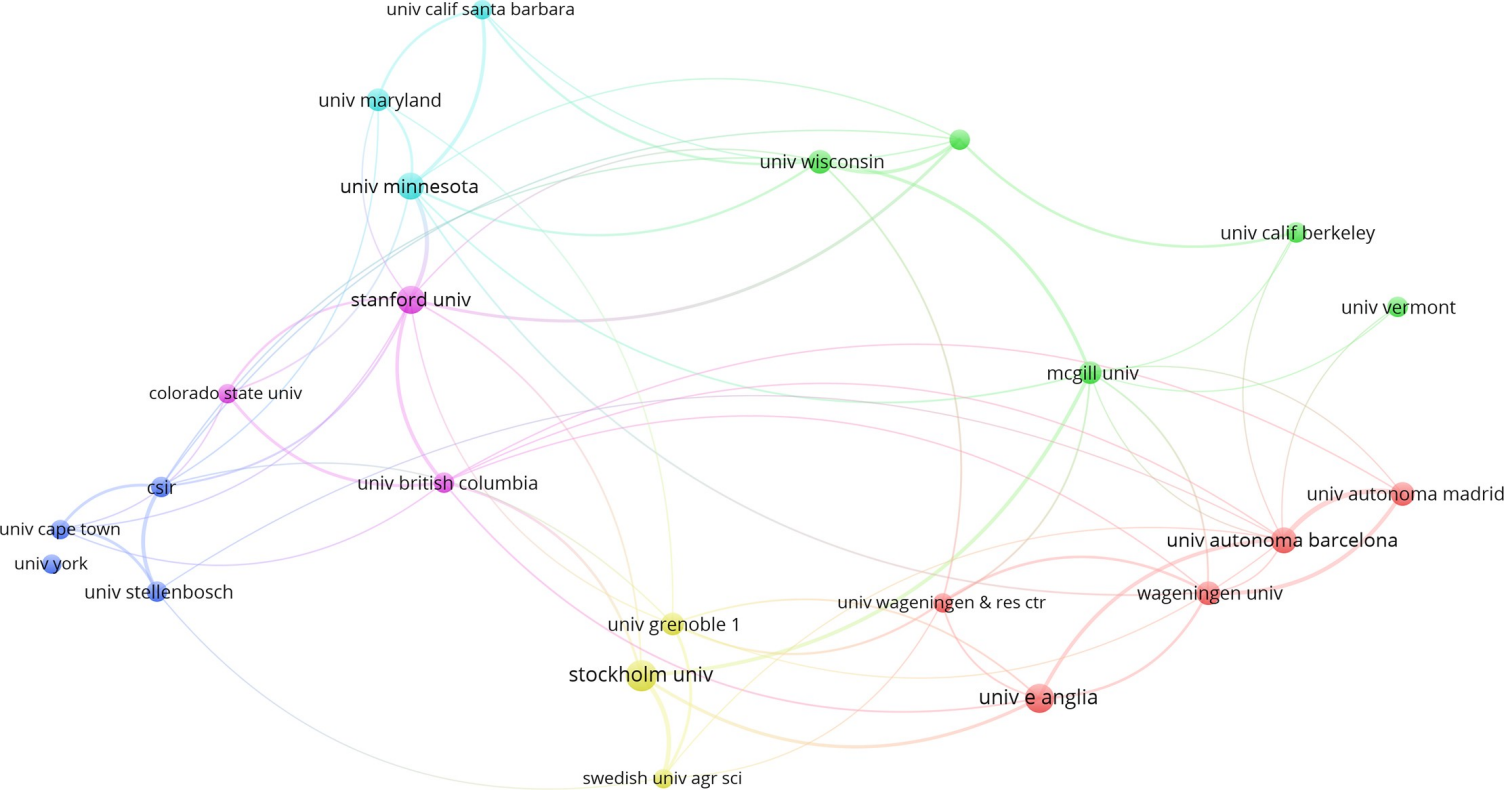
Co-authorship network between top institutions based on the total number of highly cited articles. Note: A threshold of 5 was applied for these 132 highly cited articles, which resulted in a total of 23 institutions. The bubble size refers to the total number of highly cited articles, while line thickness and color refer to link strength and clustering, respectively.

Stockholm University, the University of East Anglia, Stanford University, the University of Minnesota, and the University of Autonoma Barcelona were among the top five institutions in the world in terms of total number of highly cited articles published from 1981 to 2017 (Fig 4). Stockholm University and Stanford University were the most productive institutions in Europe and North America, respectively (Fig 4). In recent years, Stanford University has developed the InVEST model (a subset of the Natural Capital Project), which has been gaining popularity among researchers. Among the most active institutions worldwide, only two came from Africa (i.e., the University of Cape Town and Stellenbosch University), while there were none from Asia and Latin America.

North America and Europe had the top institutions in terms of co-authorship network. There were eleven institutions from North America and nine institutions from Europe. In North America, the US is the main contributor of highly cited articles relevant to ecosystem services. The results also show that nine of the most productive institutions are from the US (Fig 4). These most cooperative local institutions are an important factor in the US becoming both a highly productive and cooperative country. As the US’ most productive institution, Stanford University had the highest link strength with the University of Minnesota among all other institutions in the country. Examples of ecosystem services research programs in the US include the National Ecosystem Services Partnership, the Natural Capital Project, and the development of USEPA’s EnviroAtlas tool [25]. In terms of total co-authorship link strength, however, the top two institutions came from Europe (i.e., Stockholm University and the University of East Anglia). The other top institutions are located in other countries, i.e., the UK, Netherlands, France, Sweden, and Spain. Among European institutions, Stockholm University had a strong link strength with the Swedish University of Agricultural Sciences in Sweden. The University of Autonoma Barcelona and the University of Autonoma Madrid in Spain also had a strong network (Fig 4).

**Fig 5:**
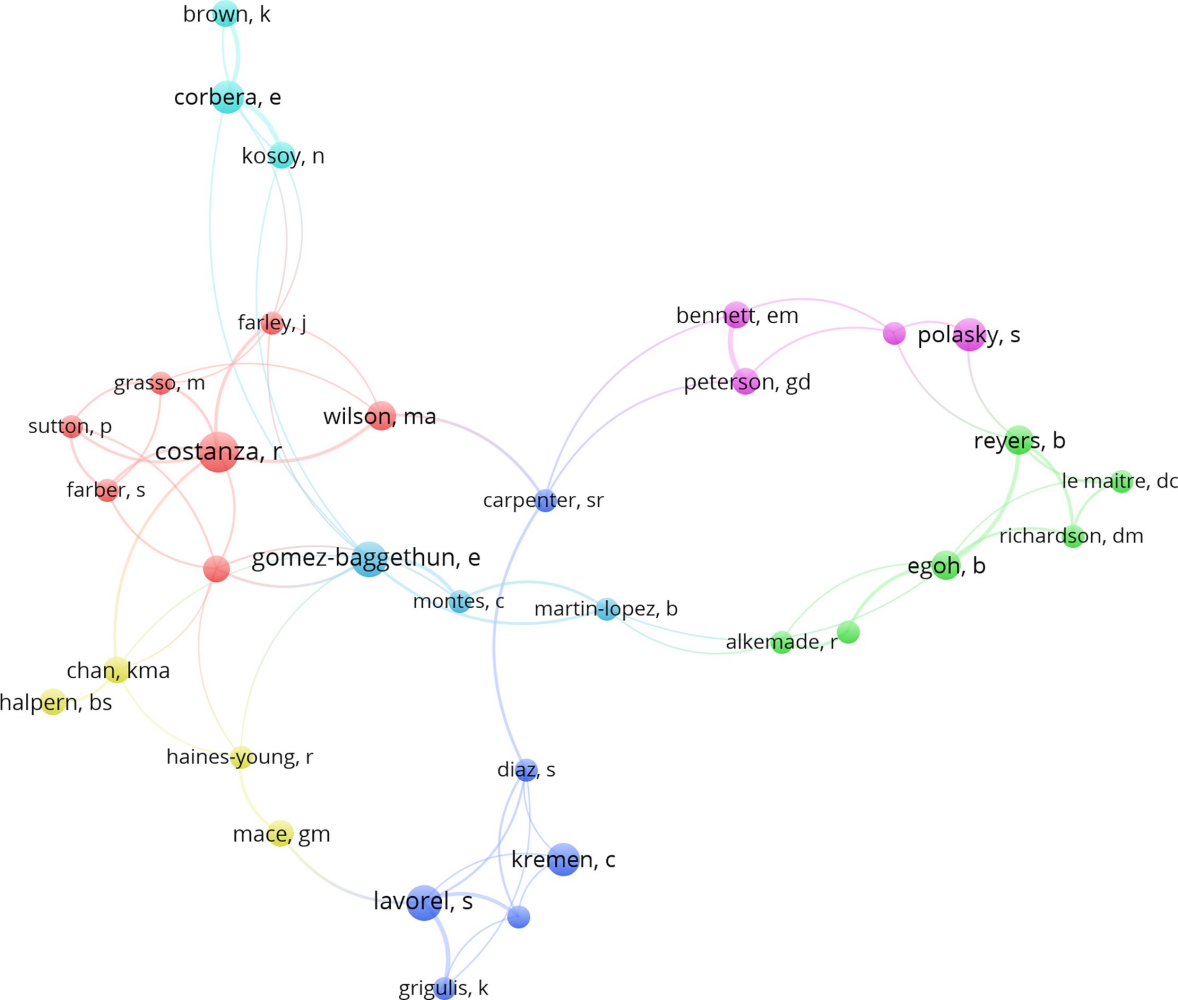
Co-authorship network of the most productive authors based on the total number of highly cited articles. Note: A threshold of 3 was applied for these 132 highly cited articles, which resulted in a total of 33 authors. The bubble size refers to the total number of highly cited articles, while line thickness and color refer to link strength and clustering, respectively.

Seven distinct clusters of co-author groupings were identified, as shown in Fig 5. Three major clusters exist in which co-authors within the clusters primarily surround one or two authors that have published a significant number of highly cited articles (e.g., those around Robert Costanza of Australian National University, Sandra Lavorel of CNRS–Université Grenoble-Alpes, and Erik Gomez-Baggethun of Norwegian University of Life Sciences). It is worthy to note that the early research on natural capital focused on economic value of ecosystem services at the global scale by Costanza et al [4]. The unit value based approach began from the study of Costanza et al [4], which has been cited approximately 6,000 times until 2017. Especially, this study established the first framework of estimating economic value of 17 ecosystem services for 16 biomes. As Costanza and Kubiszewski [26] discussed, ecosystem services involving problems and projects should also be required to address multiple disciplinary perspectives and encourage transdisciplinary cooperation.

Analysis also revealed that Robert Costanza, Sandra Lavorel, and Erik Gomez-Baggethun were the top three most productive authors in terms of the number of highly cited articles (Fig 5). Not only that these authors have strong interests in the field of ecosystem services, the results are also indicative of how influential their works are in the field. In another study, Robert Costanza was also found to be the most productive author according to the ISI Web of Science as of January 2011 [26]. His research interests include ecological economics, landscape ecology, and ecosystem services.

### Characteristic of highly cited articles on ecosystem services

**Fig 6:**
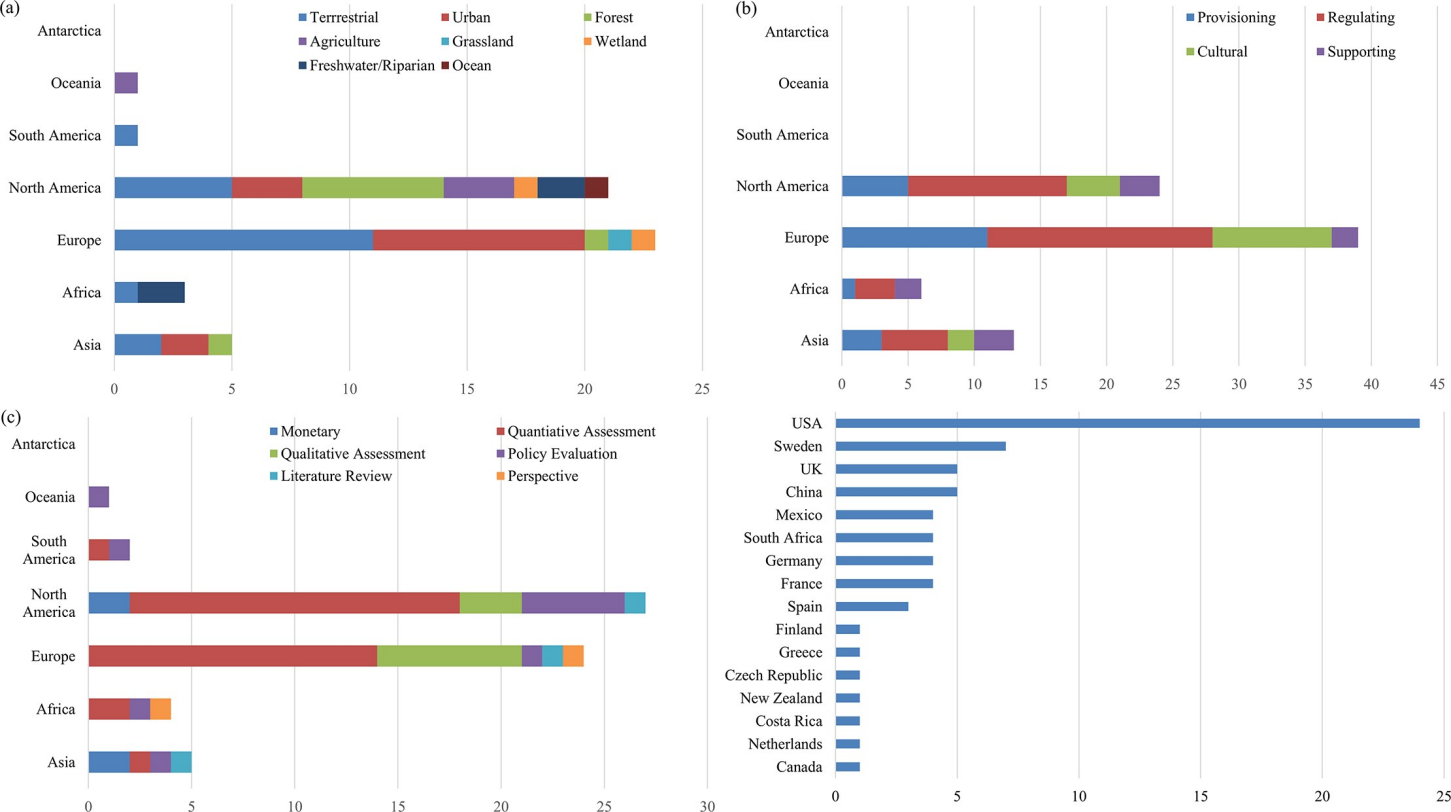
Number of highly cited articles for each (a) type of ecosystem, (b) ecosystem service, (c) research focus, and (d) case study site.

The distribution of the remaining 71 articles out of the 132 initial highly cited articles across continent of origin, ecosystem types, ecosystem services, research focus and case study site are given in Fig 6. North America and Europe were the dominant continents in terms of number of highly cited articles and number of ecosystems studied (Fig 6). The top two continents, i.e., Europe and North America, have also been highlighted in [58]. In contrast, there were only a limited number of ecosystems studied in South America and Oceania. The most assessed ecosystems were the terrestrial, urban, and forest (Fig 6(A)). More specifically, urban ecosystem was the dominant type in Europe, while forest ecosystem received special attention in North America. Overall, this analysis found very few assessments involving grassland, ocean, and wetland ecosystems among the highly cited articles. These ecosystem services will most likely receive increased attention in the next coming years due to current environmental degradation (e.g., the effect of grassland degradation on ecosystem services [59–60]).

In terms of the type of ecosystem services studied (Fig 6(B)), regulating services were the most dominant, consistent with the previous finding [15]. Most highly cited articles were conducted in the four continents, i.e., Europe, North America, Asia, and Africa, and were associated with regulating and provisioning services. The complexity of evaluating cultural and supporting services (e.g., [61–62]) might have been a factor to the relatively lower number of highly cited articles of these services. In Africa, there were no highly cited articles focusing on cultural services.

The results on research focus are given in Fig 6(C). Quantitative assessment was the top research focus with regards to studies on ecosystem services, followed by qualitative assessment, policy evaluation and monetary valuation. Quantitative assessments include all kinds of empirical investigation of ecosystem services through statistical or computational techniques [19]. The two major tools used to assess ecosystem services quantitatively are the Integrated Valuation of Ecosystem Services and Trade-offs (InVEST) and the Social Values for Ecosystem Services (SolVES) [63]. In addition to quantifying the effects of land use on multiple ecosystem services and their trade-offs [64], some case studies were conducted to visualize the relationships between multiple ecosystem services and biodiversity [65–66]. Qualitative assessments of ecosystem services have been conducted mainly through surveys, experiments, or questionnaires. Some studies have also addressed the relationship between biodiversity and ecosystem services [67–68]. Ecosystem service-related policy evaluation is also a major topic worldwide. The Payment for Ecosystem Services (PES) is gaining an increasing attention on a global scale. It has recently emerged as a policy solution for enhancing or safeguarding ecosystem service provisions [69–71]. Costa Rica pioneered the use of formal PES mechanisms in 1997 [72]. China has also implemented a number of national PES policies, including the Natural Forest Conservation Program (NFCP) and the Grain to Green Program (GTGP), which are among the largest programs of their kind in the world [73]. Neoliberal environmental policy has created a stable ecosystem services market in the US and elsewhere, which includes wetland mitigation banking [74–75]. Mexico has also become one of the world’s leading advocates of PES by jointly operating with Costa Rica through government-financed programs that are managed by a national agency (i.e., Mexico’s National Forest Commission (CONAFOR)) [76–77]. Monetary valuation approaches have also been applied for valuing ecosystem services (e.g., the unit value transfer method, contingent valuation method, and market and non-market valuation) [4, 6–7, 78–79]. Based on our assessment of highly cited articles, monetary valuations conducted on a global scale are mainly the work of Costanza [4, 6–7, 80]. It is worth noting that Costanza is the leading author of ecosystem service articles and consistently produces the highest number of highly cited articles in that field. Based on different conditions, Costanza et al. [7] estimated that the total global ecosystem services as of 2011 were worth either $125 trillion per year (assuming updated unit values and changes to biome areas) or $145 trillion per year (assuming only unit values changed). Monetary valuations were also conducted in other countries, especially in the US [81–82] and China [83–84]. These highly cited articles have probably promoted the development of ecosystem services quantification. A previous study found that the US and China are the major countries in terms of ecosystem service value mapping [58]. Additionally, more than half of ecosystem services studies in China are devoted to monetary valuation, especially based on the unit value transfer method [19].

The number of case study sites involving highly cited articles is shown in Fig 6(D). The results show that case study sites were not well distributed across continents. They were mainly located in two continents (i.e., North America (e.g., the US and Mexico) and Europe (e.g., Sweden and the UK). Most countries with a large number of case study sites (Fig 6(D)) were also the most productive countries (Fig 3). The top five countries in terms of number of case study sites were the US (18.18%), Sweden (5.3%), the UK (3.79%), China (3.79%), and Mexico (3.03%). In terms of the total number of highly cited articles, the US, Sweden, and the UK ranked 1, 5, and 2, respectively (Fig 3). The top North American countries in term of number of case study sites were the US and Mexico. The leading European countries were Sweden, the UK, and Germany. The top Asian country was China. Finally, South Africa was the leading African country. Very few case study sites were found in South America and Oceania.

### Scope and limitations of this analysis

This bibliometric analysis is based solely on the SCI-E and SSCI databases of the Web of Science. In addition, only two terms (i.e., either “ecosystem service” OR “ecosystem services”) were used as search terms to generate sample articles for the analysis. Other databases also exist. We recognize that results may differ according to database (e.g., Scopus and Google Scholar) and the inclusion of other search terms (e.g., ecosystem valuation). There were some highly cited articles published in some journals (e.g., *PLoS ONE*, *PNAS*) that did not contain author keywords. Thus, our analysis included only the articles with available author keywords for presenting the keywords network. The citation threshold (>100) was used and referred to as the highly cited articles in this study. Highly cited articles were searched for up until the end of 2017, with most being distributed between 2005 and 2014. Thus, all conclusions presented here should be interpreted within the context of these limitations.

## Summary and conclusions

In this paper, we have presented the global research trends and scholarly networks on ecosystem services covering the period 1981–2017. Our analysis included bibliometric information regarding publication outputs, journals, author keywords, countries, institutions, and authors, and was based on 132 highly cited research articles retrieved from the SCI-E and SSCI databases of the Web of Science. The types of ecosystem and ecosystem services assessed, the research focus of the studies evaluated, and the geographical location of case study sites were also considered.

A relative increase in the number of highly cited articles indicates that the ecosystem services concept has gained an increasing attention, especially since the mid-2000s. The top five author keywords were “ecosystem services,” “biodiversity,” “valuation,” “resilience,” and “conservation planning.” Based on the author keywords network, “ecosystem services” is strongly linked to biodiversity. Both important “old” (e.g., sustainability) and “new” (e.g., payment for ecosystem services) concepts are relevant in the author keywords network. The top three journals in terms of number of highly cited articles were *Ecological Economics*, *PNAS*, and *Ecological Indicators*. Notably, *Ecological Economics* contributed 27 highly cited articles, i.e. more than 20% of the total number.

Despite ranking sixth overall, *Science* ranked first in both impact factor and total citations per article. Respectively, the US, UK, Netherlands, Spain, and Sweden were the top five most productive and cooperative countries based on the total number of highly cited articles and co-authorship network. Notably, the US was highly connected to Canada, the Netherlands, China and the UK. Stockholm University and Stanford University were the most productive institutions in Europe and North America, respectively. As the most productive institution in the US, Stanford University was most strongly linked to the University of Minnesota among all other institutions in the country and Stanford University gained a lot of recognition for the InVEST model. Robert Costanza was the most prolific and highly cited author. The unit value based approach began from Costanza et al [4], and the first framework of estimating economic value of 17 ecosystem services for 16 biomes was established at the global scale. Terrestrial, urban, and forest ecosystems were the top ecosystems assessed or studied. Quantitative and qualitative assessments were the main focus of ecosystem service studies. Most studies were conducted in North America and Europe.
